# Sirt1 expression is associated with CD31 expression in blood cells from patients with chronic obstructive pulmonary disease

**DOI:** 10.1186/s12931-016-0452-2

**Published:** 2016-10-27

**Authors:** Ryo Kato, Shiro Mizuno, Maiko Kadowaki, Kohei Shiozaki, Masaya Akai, Ken Nakagawa, Taku Oikawa, Masaharu Iguchi, Kazuhiro Osanai, Takeshi Ishizaki, Norbert F Voelkel, Hirohisa Toga

**Affiliations:** 1 Department of Respiratory Medicine, Kanazawa Medical University, 1-1 Daigaku, Uchinada, Kahoku-gun, Ishikawa 920-0265 Japan; 2 Department of Respiratory Medicine, University of Fukui, Fukui, Japan; 3 Department of Respiratory Medicine, Japanese Red Cross Fukui Hospital, Fukui, Japan; 4 Pulmonary and Critical Care Medicine Division, Virginia Commonwealth University, Richmond, VA USA

**Keywords:** miR-34a, miR-126, p53, Sirt1

## Abstract

**Background:**

Cigarette smoke induced oxidative stress has been shown to reduce silent information regulator 1 (Sirt1) levels in lung tissue from smokers and patients with COPD patients. Sirt1 is known to inhibit endothelial senescence and may play a protective role in vascular cells. Endothelial progenitor cells (EPCs) are mobilized into circulation under various pathophysiological conditions, and are thought to play an important role in tissue repair in chronic obstructive lung disease (COPD). Therefore, Sirt1 and EPC-associated mRNAs were measured in blood samples from patients with COPD and from cultured CD34^+^ progenitor cells to examine whether these genes are associated with COPD development.

**Methods:**

This study included 358 patients with a smoking history of more than 10 pack-years. RNA was extracted from blood samples and from CD34^+^ progenitor cells treated with cigarette smoke extract (CSE), followed by assessment of CD31, CD34, Sirt1 mRNA, miR-34a, and miR-126-3p expression by real-time RT-PCR.

**Results:**

The expression of CD31, CD34, Sirt1 mRNAs, and miR-126-3p decreased and that of miR-34a increased in moderate COPD compared with that in control smokers. However, no significant differences in these genes were observed in blood cells from patients with severe COPD compared with those in control smokers. CSE significantly decreased Sirt1 and increased miR-34a expression in cultured progenitor cells.

**Conclusion:**

Sirt1 expression in blood cells from patients with COPD could be a biomarker for disease stability in patients with moderate COPD. MiR-34a may participate in apoptosis and/or senescence of EPCs in smokers. Decreased expression of CD31, CD34, and miR-126-3p potentially represents decreased numbers of EPCs in blood cell from patients with COPD.

## Background

Cigarette smoke-induced oxidative stress is a major risk factor for chronic obstructive pulmonary disease (COPD). Numerous studies have indicated the presence of endothelial dysfunction in smokers and in patients with COPD [1, 2], and have also suggested that reduced numbers and/or dysfunction of endothelial progenitor cells (EPCs) could contribute to the tissue angiogenesis and repair mechanisms [3–6]. EPCs, first reported by Asahara et al. [7], play a key role in angiogenesis and in the maintenance of vascular integrity [8, 9] and also have the potential to maintain lung structure in smokers [10]. EPCs are bone marrow-derived endothelial cell precursors that can migrate to sites of neovascularization, differentiate into endothelial cells, and participate in angiogenesis and hypothetically in the maintenance of stressed lung microcirculation [8]. Analysis of a combination of stem cell and endothelial cell markers is most commonly applied to isolate EPCs from blood cells [11]. The cluster of differentiation (CD) 34, also known as the hematopoietic progenitor cell antigen, is a member of a family of single-pass transmembrane sialomucin proteins expressed on early hematopoietic and vasculature-associated tissues [12]. CD34^+^ progenitor cells differentiate into EPCs, which express surface markers for endothelial cells, such as CD31, vascular endothelial growth factor receptor 2. CD31, also known as the platelet endothelial cell adhesion molecule, is mainly expressed on endothelial cells, platelets, and macrophages and is thought to be an immature and mature vascular endothelial cell marker [8].

Micro RNAs (miRNAs) are non-coding RNA molecules that modulate gene expression by binding to their complementary sequences on target mRNAs; they can regulate cell proliferation, differentiation and apoptosis [13–15]. Recently, the possible involvement of two miRNAs, miR-34a and miR-126, in the regulation of EPCs has been supported by animal studies using silencing of their target genes in hematopoietic stem cells [16, 17]. The miRNAs in blood cells may affect EPC activity and perhaps the lung maintenance program and pathogenesis in COPD. MiR-34a is the most intensely investigated miRNA that is induced by p53 and its expression is closely related to the induction of apoptosis and cell cycle arrest in cancer cells [18]. As an NAD-induced deacetylase, silent information regulator 1 (Sirt1) is a transcriptional regulator, that can suppress the expression of pro-apoptotic proteins [19], and regulates p53-dependent apoptosis by deacetylating and destabilizing the p53 protein. It is known that Sirt1 mediates miR-34a-induced apoptosis by regulating p53 activity, and a positive feedback loop has been identified, wherein p53 induces miR-34a expression, thus suppressing Sirt1 and increasing p53 activity [20]. We have previously shown that miR-34a is associated with p53 protein expression, both in animal emphysema models and in human COPD lung tissues [21–23], suggesting that miR-34a, regulated by p53, is also involved in lung tissue remodeling via the p53 pathway. In the context of EPCs and miRNA, Zhao et al., reported that miR-34a impairs EPC-mediated angiogenesis by inhibiting Sirt1 in rats [16], indicating that oxidative stress-induced p53 and miR-34a expression could induce vascular endothelial cell senescence and decrease EPC activity through Sirt1 inhibition. MiR-126 has been identified as the miRNA, which is highly expressed in endothelial cells [24, 25] and also detected in bone marrow derived cells [17]. MiR-126 plays a critical role in the regulation and development of endothelial differentiation and vascular remodeling [26]. The precursor miRNA (pre-miR-126) produces two mature strands, miR-126-3p and miR-126-5p [24]; administration of miR-126-3p has been shown to promote the incorporation of Sca-1^+^ progenitor cells [27], suggesting an essential role for miR-126 in the endothelial stress response and the release of EPCs.

Here we investigated whether expression of vascular endothelial cell genes expression in blood cells from smokers is associated with the development of COPD. We first analyzed the CD31 and CD34 mRNA expression in blood cells obtained from 358 smokers to explore the association of airflow limitation and EPC associated gene expression. We then measured Sirt1 mRNA, and miR-34a and miR-126 expression to clarify this association and the regulation of oxidative stress in blood cells, cultured CD34^+^ progenitor cells, and in cultured pulmonary endothelial cells. We speculated that gene expression in blood cells could be a biomarker representing the numbers of circulating EPCs or the dysfunction of EPCs from smoking-induced oxidative stress, which may be a marker of effective lung tissue maintenance status.

## Methods

### Patients

From October 2011 to December 2014, 358 patients were recruited from the outpatient department of the Division of Respiratory Medicine, Kanazawa Medical University Hospital, Department of Respiratory Medicine, University of Fukui Hospital, and the Division of Respiratory Medicine, Fukui Red Cross hospital. The inclusion criteria for enrollment were age >40 years and at least a 10 pack-year history of tobacco exposure. The study was approved by the Research Ethics Committee of Kanazawa Medical University, University of Fukui, and Fukui Red Cross Hospital (Protocol: NO. 0073). All subjects gave informed consent in writing.

Pulmonary function tests were performed to determine FVC and FEV1. Diagnosis of COPD was based on the basis of clinical history, physical examination, and spirometric data, following the Global Initiative for Obstructive Lung Disease (GOLD) classification [28]. This study included 252 patients with COPD patients and 106 smokers without COPD. The COPD patients were classified into 3 categories (mild; GOLD I, moderate; GOLD II, severe; GOLD III and IV) based on spirometric data (Table 1).

**Table 1:** Characteristics of patients

Characteristics	Smoking control	Mild COPD	Moderate COPD	Severe COPD
GOLD	-	Stage I	Stage II	Stage III, IV
Number of patients	106	69	103	80
Age (years)	70.5 ± 9.4	72.2 ± 9.3	72.5 ± 8.8	73.9 ± 8.7
Gender (Male/Female)	(102/4)	(65/4)	(98/5)	(76/4)
FVC predict (%)	97.2 ± 14.2	114.1 ± 14.9*	97.3 ± 16.1	75.7 ± 18.1*
FEV1/FVC (%)	77.3 ± 6.1	64.5 ± 5.1*	52.8 ± 9.1*	39.3 ± 10.4*
FEV1% predict	96.1 ± 13.5	96.3 ± 13.3	64.5 ± 8.8*	37.5 ± 8.1*
LAA (%)	5.5 ± 6.1	10.7 ± 10.0	14.6 ± 10.7*	22.7 ± 14.6*
Smoking (Pack years)	61.4 ± 38.5	58.1 ± 33.7	60.5 ± 30.7	63.2 ± 29.2
Current smoker (no.)	31	22	29	19
Medication (no.)				
LABA	-	17	49	50
LAMA	-	17	46	55
ICS	-	11	36	38
CRP (mg/dl)	0.24 ± 0.34	0.31 ± 0.48	0.39 ± 0.77	0.46 ± 0.92

*COPD* Chronic obstructive pulmonary disease, *GOLD* Global Initiative for Obstructive Lung Disease, *FEV1 % predict* Forced Expiratory Volume in 1 s % predicted, *LAA* Low attenuation area; change in FEV1 as a percent of baseline FEV1, *LABA* Long acting beta 2 agonist, *LAMA* Long acting muscarinic antagonist, *ICS* Inhaled corticosteroid

Values are expressed as means ± SD. * *p* < 0.05 vs smoking control

CT scans were acquired using either a 64 or a 128 multidetector CT scanner (Somatom Definition FLASH or Somatom Definition AS+; Siemens Medical Solutions, Erlangen, Germany; Brilliance 64, Phillips, Eindhoven, Netherlands) with a slice thickness of less than 2 mm. We calculated the % of low attenuation area (%LAA) using a threshold of −960 HU to assess the emphysematous changes and the total lung volume using a computer software LungVision™ version 2.1(Cybernet Systems CO. LTD., Tokyo, Japan.).

### Generation of Cigarette Smoke Extract (CSE)

CSE was prepared as reported previously [29]. Briefly, one cigarette without filters (Marlboro; Philip Morris International Inc; New York City, New York) was burned, and the smoke was passed, using a vacuum pump, through a glass Cambridge filter (Cambridge Filter Japan, Ltd; Tokyo, Japan) with 0.20-μm pores for removing the particles and bacteria into a vessel containing phosphate-buffered saline (PBS) (1 mL per one cigarette). The CSE-PBS solution was freshly prepared for each set of experiments.

### Cell culture

Human CD34^+^ progenitor cells and human pulmonary microvascular endothelial cells (HPMVECs) were purchased from Lonza. Human CD34^+^ progenitor cells were cultured in hematopoietic progenitor cell expansion medium supplemented with a cytokine mix (PromoCell). HPMVECs were cultured in endothelial cell growth medium supplemented with 5 % FBS (Lonza). The cells were cultured in 175-cm^2^ tissue culture flasks and maintained in a cell-culture incubator (37 °C, 5 % CO_2_, and 95 % air) and then used for all the experiments. After incubation, the human CD34^+^ progenitor cells were harvested and seeded in 96-well culture plates with or without various concentrations of CSE for the MTS assay. Human CD34^+^ progenitor cells and HPMVECs were seeded in 24-well culture plates with or without 0.3 % CSE for RT-PCR analysis. HPMVECs were seeded in 6-cm culture dishes with or without 0.3 % CSE for western blot analysis, and cultured for 24 h.

### MTS assay in CD34^+^ progenitor cells

MTS assay was performed using a CellTiter 96® AQueous One Solution Cell Proliferation Assay kit in accordance with the manufacturer’s protocol. Briefly, human CD34^+^ progenitor cells were seeded in 96-well culture plates and cultured with or without various concentrations (0.1 to 3 %) of CSE. The cells were then incubated with 20 μL of CellTiter 96® AQueous One Solution Reagent for 15 min, and absorbance at 490 nm was measured.

### Real-time RT-PCR analysis of mRNA and miRNA

Whole blood was collected from each patient into PAXgene Blood RNA Tubes (PreAnalytiX) using standard phlebotomy technique and stored at −20 °C until used for RNA extraction. RNA from the blood was extracted using the PAXgene Blood RNA Kit (PreAnalytiX) according to the manufacturer’s protocol. Isolation of total RNA and miRNA from CD34^+^ progenitor cells and HPMVECs was performed using an miRNeasy Mini kit according to the manufacture’s protocol. For qPCR, the SuperScript VILO cDNA kit (Life Technologies) was used for mRNA analysis, and the miScript II RT kit (Qiagen) was used for miRNA analysis. As per the manufacturer’s instructions, 0.5 μg RNA per sample in 20 μL and the cDNA was diluted 1:10 prior to PCR. PCR was performed with the cDNA using specific oligonucleotide primers. PCR was performed in duplicate on a LightCycler™ PCR system (Roche Diagnostics, Meylan, France) using the DNA binding SYBR Green dye (Roche Diagnostics) for mRNA analysis and the miScript SYBR Green dye (Qiagen) for miRNA analysis and detection of PCR products. The 18 s rRNA gene was used as the reference for mRNA, and miR-103 was used as the reference for miRNA. The sequence of the target primers is shown in Table 2.

**Table 2:** Primer sequences used for real-time quantitative PCR of mRNA and miRNA

Target gene	Upper-primer	Lower-primer
CD31	5′-AACCCACTCCCCGACCTAGA-3′	5′-CCAGACACCATTCCAAAACC-3′
CD34	5′-TAAGAAGGACAGGGGAGAGG-3′	5′-GCCAAGACCAGCAGTAGACA-3′
Sirt1	5′-GGGGTGTCTGTTTCATGTGG-3′	5′-ACATCGCTTGAGGATCTGGA-3′
IL-6	5′-GGTACATCCTCGACGGCATC-3′	5′-TGCCTCTTTGCTGCTTTCAC-3′
p21	5′-GGAAGACCATGTGGACCTGT-3′	5′-GGCGTTTGGAGTGGTAGAAA-3′
18 s rRNA	5′-GACTCAACACGGGAAACCTC-3′	5′- CGCTCCACCAACTAAGAACG-3′
miR-34a	5′-GGCAGTGTCTTAGCTGGTTGT-3′	
miR-126-3p	5′-TCGTACCGTGAGTAATAATGC-3′	
miR-103	5′-CAGCATTGTACAGGGCTATGA-3′	

### Western blot analysis

Cytoplasmic and nuclear proteins from HPMVECs were prepared using the NE-PER Nuclear and Cytoplasmic Extraction Reagents according to the manufacturer’s protocol, and the protein extracts were analyzed for protein content using the Bradford method. Each sample was quantified, and 40 μg of protein (cytoplasmic protein) or 20 μg of protein (nuclear protein) was loaded into each lane of a 4–12 % Bis-Tris Nupage gel with MES SDS running buffer, according to the manufacturer’s protocol. After electrophoresis, the proteins were transferred to a PVDF membrane, and the membrane was probed with specific primary and secondary antibodies (Santa Cruz Biotechnology Inc.). The ECL system was used for detecting of the proteins. Lamin B protein was used as the reference protein for Sirt1, and ß-actin was used as the reference protein for p53 protein.

### Measurement of Sirt1 protein expression in blood cells from COPD patients

Whole blood was collected from 48 patients (Table 3) into 6 ml round-bottom tubes containing EDTA, nucleated cells were separated from red blood cells using Hetasep™ (Stemcell Technologies) according to the manufacture’s protocol, then the pellets of blood cells were stored at −80 °C until measurement of Sirt1 protein expression. The Sirt1 protein expression of the stored blood cells was measured using a human Sirt1 ELISA kit (Abcam). The Sirt1 protein expressions in blood cells were referenced to the protein concentration measured by the Bradford method.

**Table 3:** Characteristics of Patients who measured Sirt1 protein expression

Characteristics	Smoking control	Mild COPD	Moderate COPD	Severe COPD
GOLD	-	Stage I	Stage II	Stage III, IV
Number of patients	12	5	20	11
Age (years)	69.6 ± 6.4	74.2 ± 5.5	75.9 ± 7.7	74.2 ± 7.6
Gender (Male/Female)	(12/0)	(4/1)	(19/1)	(10/1)
FVC predict (%)	99.2 ± 15.6	114.1 ± 5.4	97.2 ± 11.6	81.9 ± 17.5*
FEV1/FVC (%)	77.1 ± 7.6	59.4 ± 7.6*	51.0 ± 9.4*	32.7 ± 6.7*
FEV1% predict	96.6 ± 14.3	88.2 ± 8.0	63.5 ± 7.3*	34.2 ± 9.2*
Smoking (Pack years)	48.1 ± 31.7	43.0 ± 11.8	56.3 ± 31.7	68.3 ± 32.9
Current smoker (no.)	3	0	6	0
Medication (no.)				
LABA	-	4	17	10
LAMA	-	3	16	8
ICS	-	2	6	7

Values are expressed as means ± SD. *p < 0.05 vs smoking control

### Statistical analysis

Age, smoking index expressed as pack-years, and pulmonary function parameters were compared using the Mann–Whitney *U* test. Analysis of protein and gene expression was performed using ANOVA with Turkey’s multiple comparisons. Correlations were analyzed by the Pearson correlation coefficient. Comparisons of circulating gene expression between current smokers and ex-smokers in severe COPD were performed using the Student’s *t*-test. Comparisons were considered statistically significant at *P* < 0.05.

## Results

### Patient characteristics

Age, gender, smoking history, pulmonary function data, %LAA and serum CRP data for patients with of COPD and control smoker subjects are summarized in Table 1. No significant differences were observed in smoking history (pack-years) between the groups, however, the proportion of current smokers among patients with severe COPD was relatively lower when compared with those among smoking controls. The population of patients receiving inhaled corticosteroids (ICS) was relatively greater among patients with severe COPD than those among other groups. The average age and CRP of patients with severe COPD was slightly greater when compared to those in smoking controls, however, there were no significant differences between the groups. The percent FVC was significantly increased in mild COPD and was decreased in severe COPD compared with that in smoking control subjects. The percent LAA was significantly increased in patients with mild, moderate, and severe COPD compared with that in smoking control subjects. The %LAA was significantly higher in patients with moderate and severe COPD compared with that in smoking control subjects, and was negatively correlated (*R* = 0.458) with the %FEV1.

### Expression of circulating endothelial cell gene expressions in smokers

**Fig. 1:**
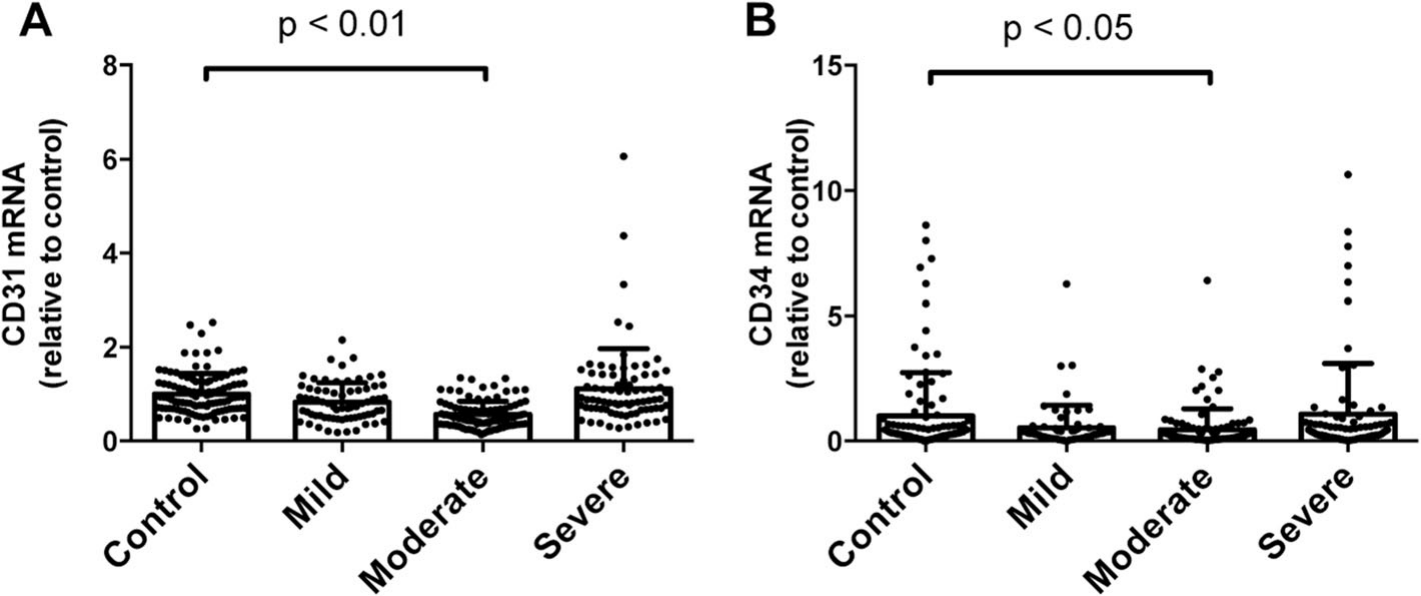
CD31 and CD34 gene expressions in blood samples from smokers. The bar graphs show data from RT-PCR analysis of CD31 (**a**) and CD34 (**b**) mRNA expressions in blood samples from smokers. The CD31 and CD34 mRNA expression were significantly decreased in subjects with moderate COPD compared to that in smoking control subjects. Data are expressed as mean ± SD

The expression of the circulating EPC genes (CD31 and CD34 mRNA) was significantly decreased in patients with moderate COPD when compared with that in smoking control subjects. Surprisingly the expression of these genes was higher in patients with severe COPD, but no significant changes were observed compared with the smoking controls (Fig. 1).

**Fig. 2:**
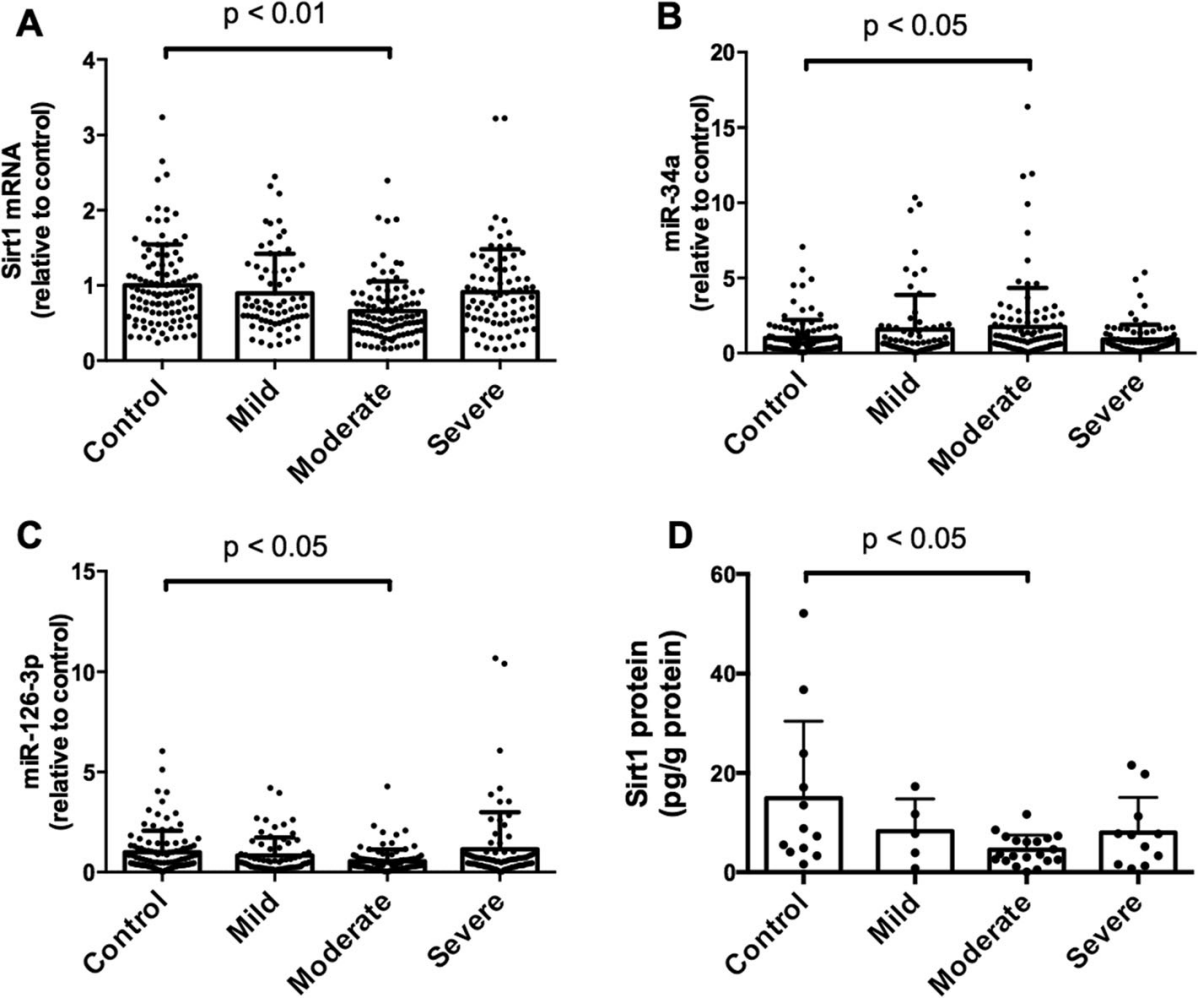
Expressions of Sirt1, miR-34a and miR-126-3p in the blood samples from smokers. The bar graphs show data from RT-PCR analysis of Sirt1 mRNA (**a**), miR-34a (**b**), miR-126-3p (**c**) expressions, and Sirt1 protein expression (**d**) in the blood samples from smokers. MiR-34a expression was significantly increased and miR-126-3p and Sirt1 mRNA and protein expression was decreased in subjects with moderate COPD compared to that in smoking control subjects. Data are expressed as mean ± SD

**Fig. 3:**
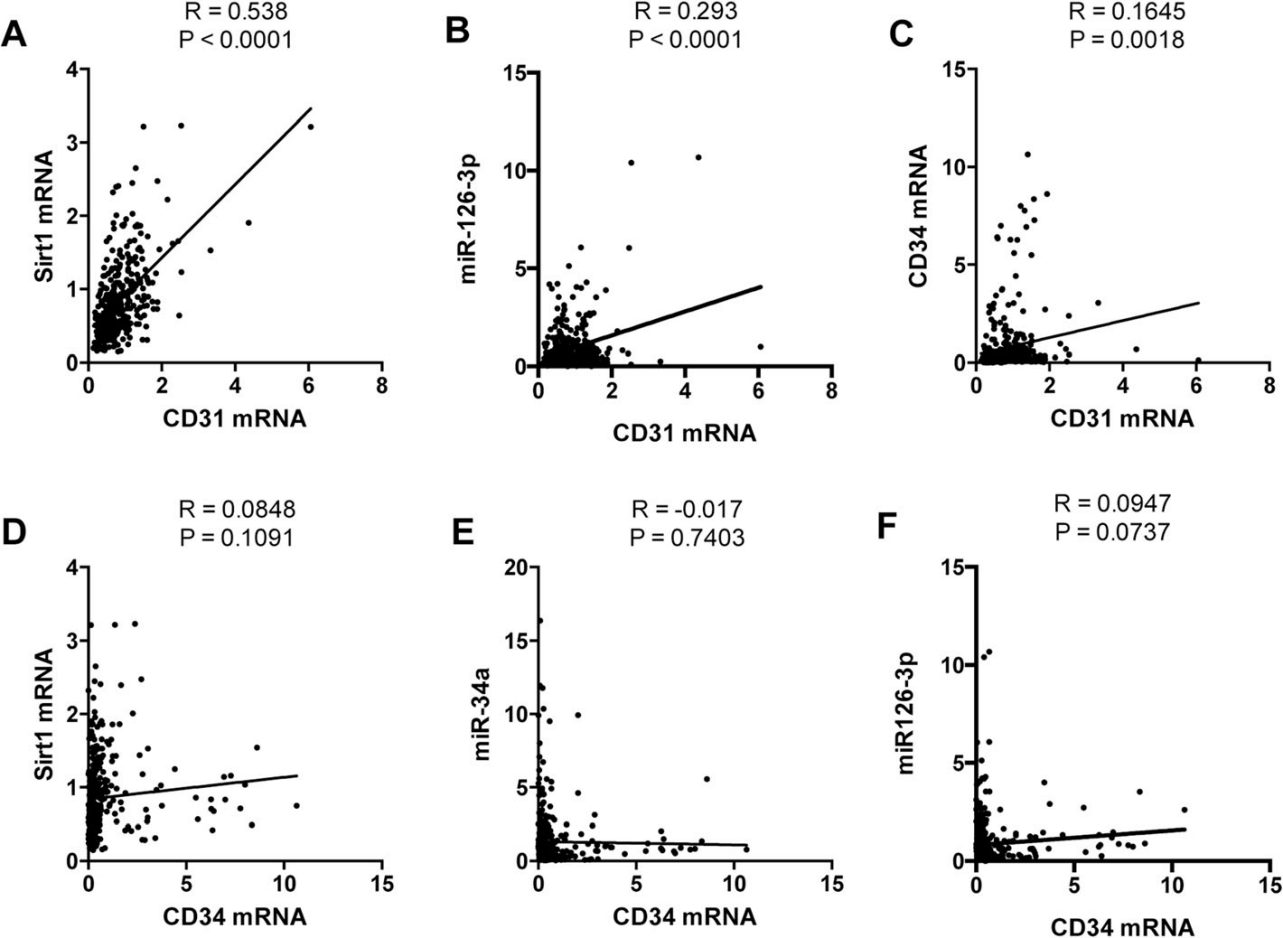
Correlation analysis between EPC related gene expressions in the blood samples from smokers. A strong positive correlation was seen between CD31 and Sirt1 mRNA (**a**), and a weak positive correlation was seen between CD31 mRNA and miR126-3p expression (**b**). A very weak positive correlation was seen between CD31 and CD34 mRNA expressions (**c**). There were no significant correlations between the expression of CD34 and Sirt1 mRNA (**d**), miR-34a (**e**), and miR-126-3p (**f**). Data are expressed as mean ± SD

Expression of miR-34a in subjects with moderate COPD was significantly increased compared to that in control subjects, and expression of miR-126-3p and Sirt1 mRNA in subjects with moderate COPD was decreased when compared with that in control subjects (Fig. 2a, b, c). A strong positive correlation was observed between the expression of Sirt1 and CD31 mRNA expression (Fig. 3a), and a weak positive correlation was observed between the expression of CD31 mRNA and miR-126-3p (Fig. 3b). We also found a very weak correlation between the expression of CD31 and CD34 mRNA (Fig. 3c). However, no significant correlation was found between CD34 expression and that of Sirt1 mRNA, miR-34a, or miR-126-3p (Fig. 3d, e, f). Additionally, we measured the blood cell Sirt1 protein expression in patients with moderately severe COPD and found it decreased when compared with the expression in the control subjects (Fig. 2d).

**Fig. 4:**
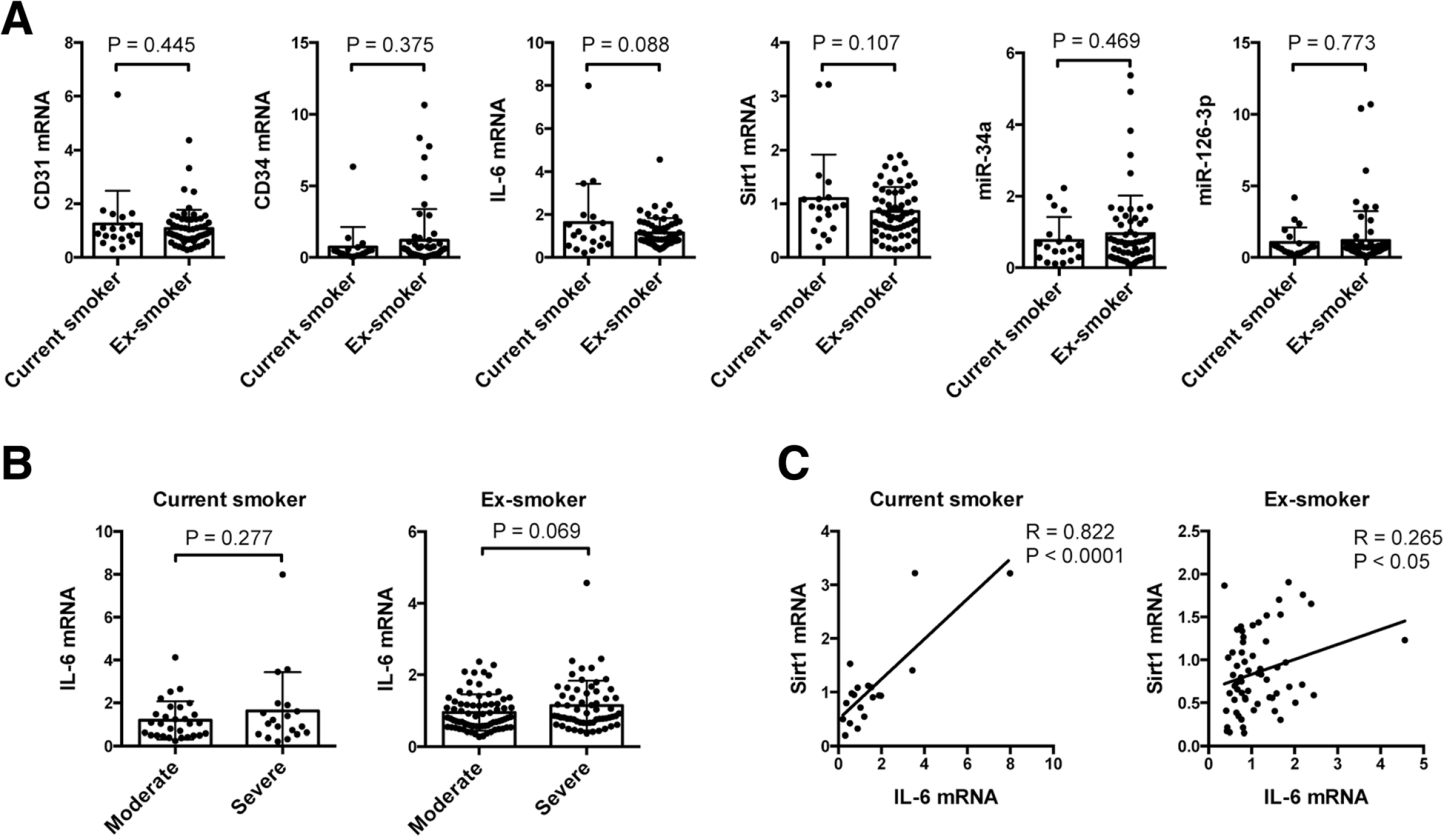
Gene expression in blood samples from patients with moderate and severe COPD between current smokers and former smokers. **a** The graphs show mean and individual data from the RT-PCR analysis of CD31, CD34, IL-6, Sirt1 mRNA, miR-34a, and miR-126-3p expressions in blood cells between current smokers and former smokers (Ex-smokers) in subjects with severe COPD. We observed a tendency toward decreased IL-6 expression in ex-smokers compared to that in current smokers. **b** The graphs show mean and individual data from RT-PCR analysis of IL-6 mRNA expression in blood cells between current smokers and ex-smokers in subjects with moderate and severe COPD. **c** A strong positive correlation was seen between IL-6 and Sirt1 mRNA expression among current smokers with severe COPD. A weak positive correlation was seen between IL-6 and Sirt1 mRNA expression among ex-smokers with severe COPD. Data are expressed as mean ± SD

**Fig. 5:**
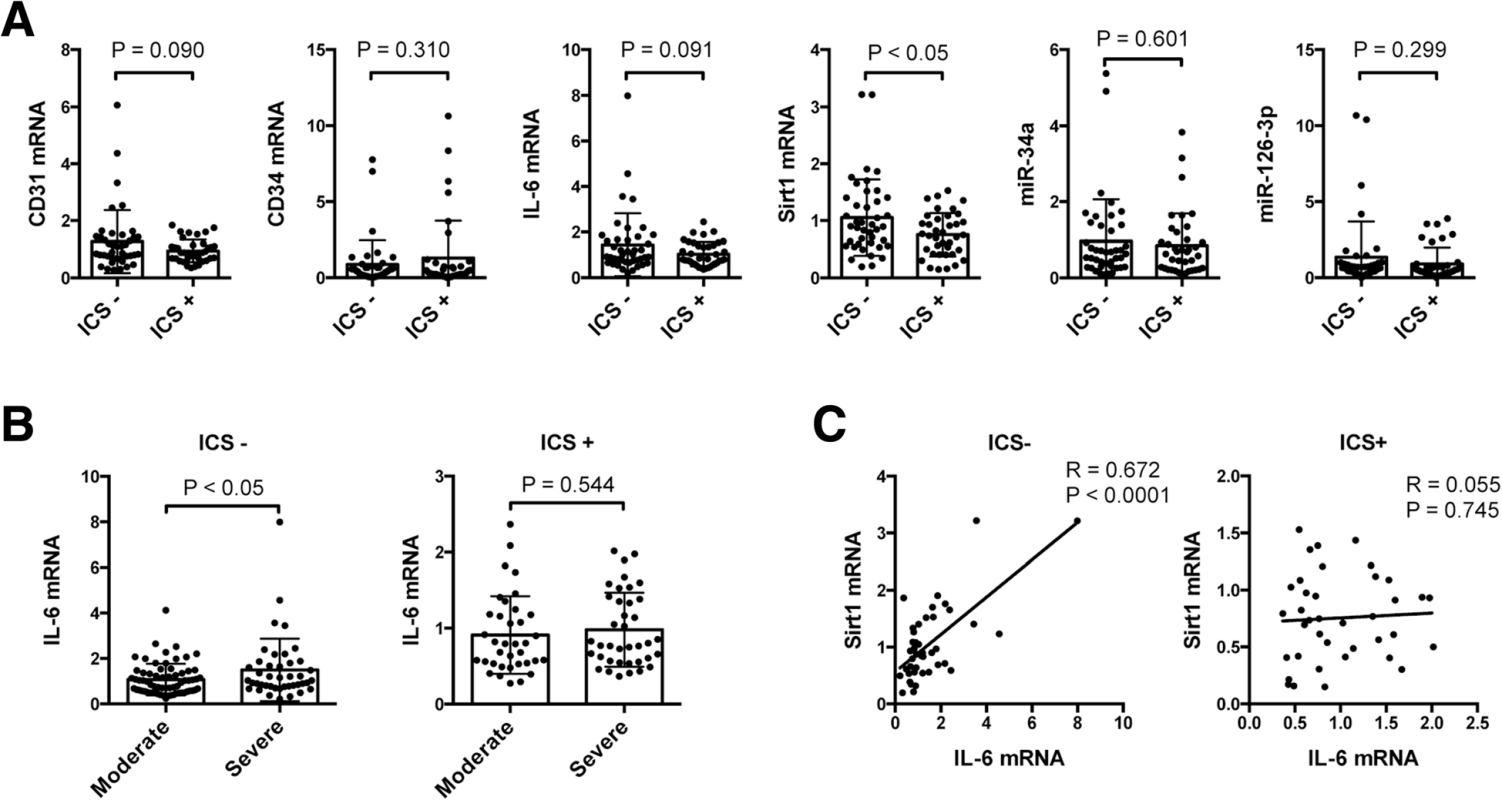
Gene expression in blood samples from patients with moderate and severe COPD between patients without ICS treatment and patients receiving ICS. **a** The graphs show mean and individual data from RT-PCR analysis of CD31, CD34, IL-6, Sirt1 mRNA, miR-34a, and miR126-3p expression between patients without ICS treatment (ICS-) and patients receiving ICS (ICS +) in subjects with severe COPD. We found a significant decrease in Sirt1 expression, and a tendency toward decreased CD31 and IL-6 expression in blood cells from patients receiving ICS. **b** The graphs show mean and individual data from RT-PCR analysis of IL-6 mRNA expression between patients without ICS treatment (ICS-) and patients receiving ICS (ICS +) in subjects with moderate and severe COPD. **c** A positive correlation was seen between IL-6 and Sirt1 mRNA expression among patients without ICS treatment (ICS -) in severe COPD subjects. No significant correlation was seen between IL-6 and Sirt1 mRNA expression among patients receiving ICS (ICS +) in subjects with severe COPD. Data are expressed as mean ± SD

Because of the smaller population of current smokers among the patients with severe COPD, we further analyzed circulating gene expression in these patients between former smokers (ex-smokers) and current smokers. Although some ex-smokers showed high expressions of CD34 mRNA, miR-34a, and miR126-3p, we did not find significant differences in the expression of these genes between current smokers and ex-smokers. However, a tendency toward increased IL-6 mRNA expression was observed in current smokers among patients with severe COPD (Fig. 4a). We also found a tendency toward increased IL-6 expression in patients with severe COPD compared with that in patients with moderate COPD among ex-smokers (Fig. 4b), and a strong positive correlation was observed between IL-6 and Sirt1 mRNA expression in currently smoking patients with severe COPD (Fig. 4c). Subsequently, to exclude the possibility that ICS therapy in severe COPD could affect the number of EPCs, we also analyzed the circulating blood cell gene expression in patients with severe COPD between patients receiving ICS and those not treated with ICS. Indeed, we found a significant decrease in Sirt1 expression and a tendency toward decreased expression of CD31 and IL-6 in patients receiving ICS (Fig. 5a). We also found significantly increased levels of IL-6 mRNA expression in patients with severe COPD compared to those with moderate COPD among patients who were not treated with ICS (Fig. 5b). A positive correlation was observed between the IL-6 and Sirt1 mRNA expression in patients with severe COPD who were not treated with ICS (Fig. 5c).

### Effects of CSE on cultured CD34^+^ progenitor cells and HPMVECs

**Fig. 6:**
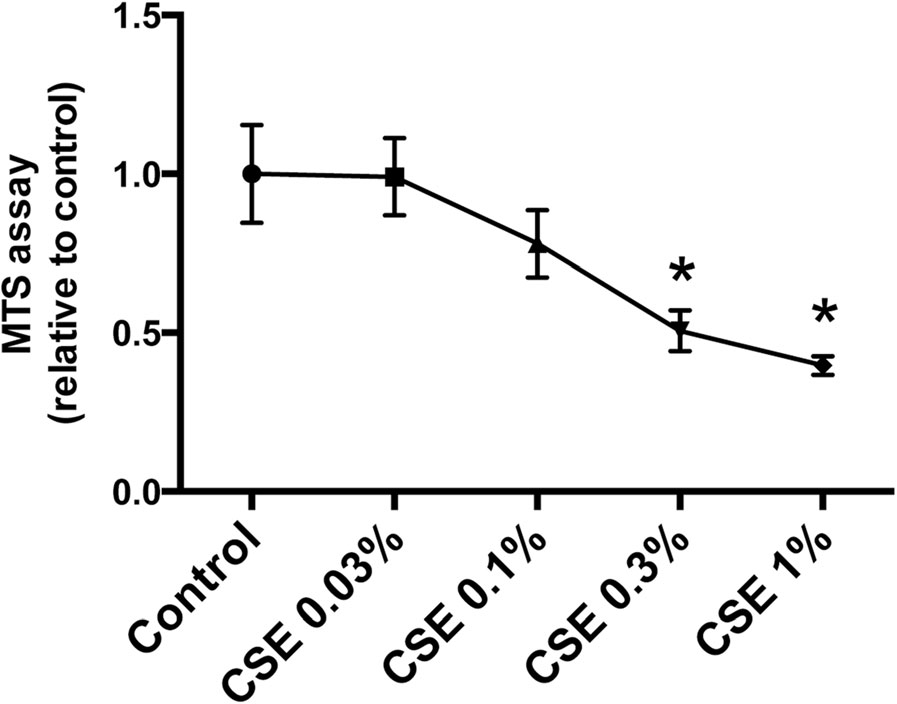
Effect of CSE on cell proliferation in cultured CD34^+^ progenitor cells. Cultured CD34^+^ progenitor cells were exposed to various concentrations of cigarette smoke extract (CSE) for 24 h, and an MTS assay was performed. CSE significantly suppressed cell proliferation at a concentration of 0.3 %. Data are expressed as mean ± SD (*n* = 8). *P < 0.05 versus control

**Fig. 7:**
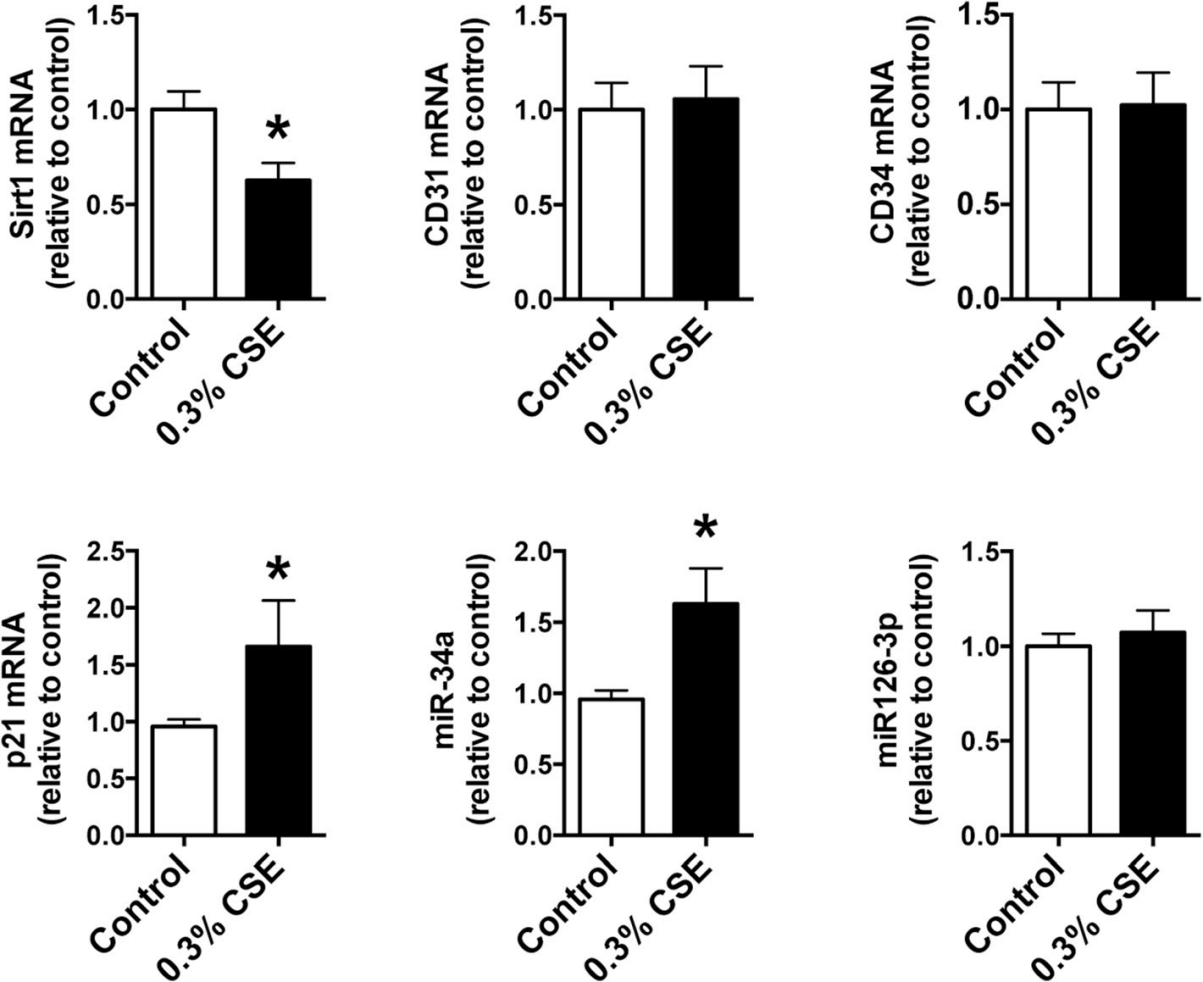
Effect of CSE on gene expressions in cultured CD34^+^ progenitor cells. Cultured CD34^+^ progenitor cells were exposed to 0.3 % of CSE for 24 h, and Sirt1, CD31, p21 mRNA, miR-34a, and miR-126-3p expression was measured by RT-PCR analysis. CSE treatment significantly suppressed Sirt1 expression and increased the expression of p21 mRNA and miR-34a. Data are expressed as mean ± SD (*n* = 6). *P < 0.05 versus control

**Fig. 8:**
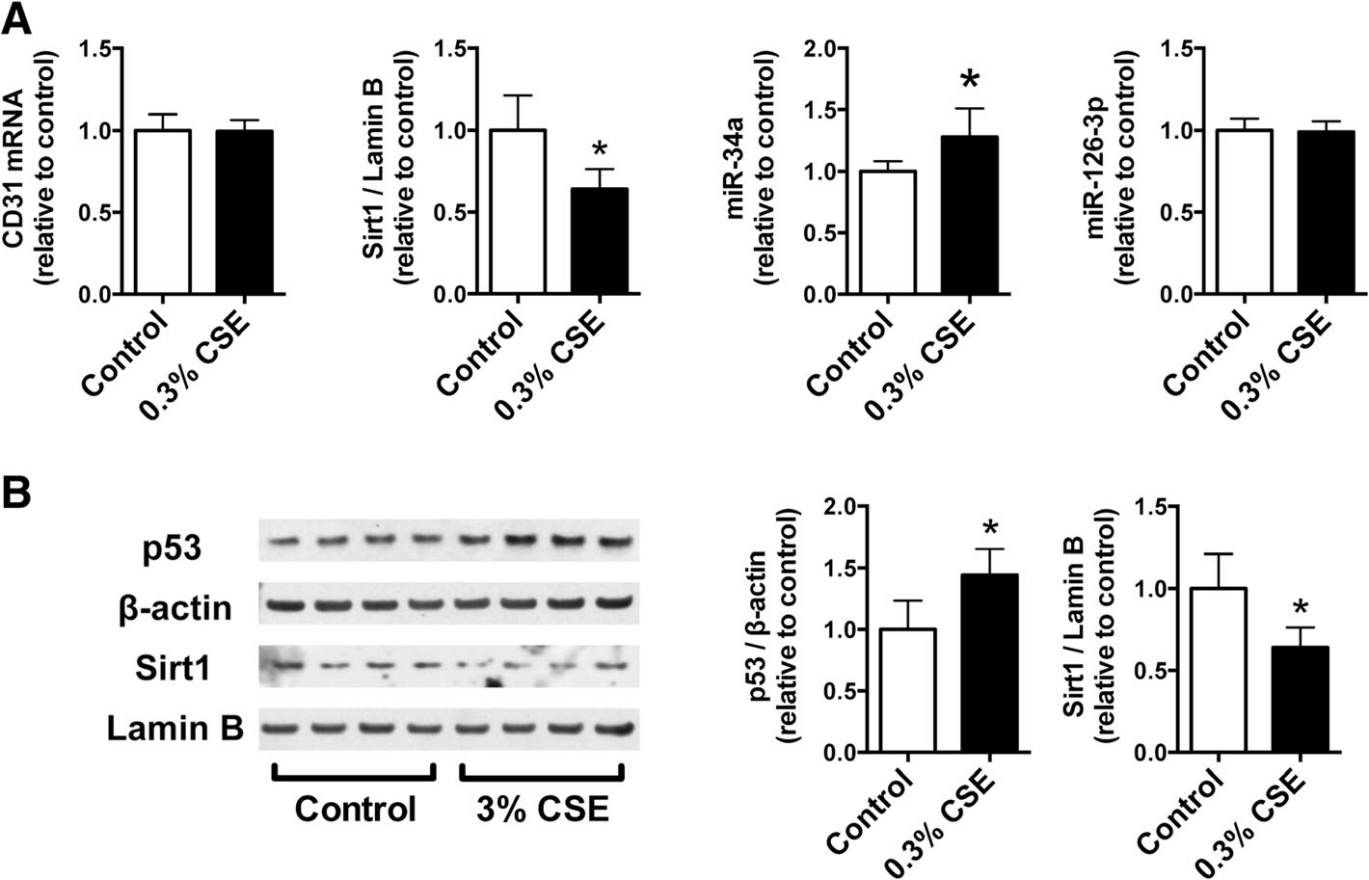
Effect of CSE on gene and protein expression in cultured human pulmonary microvascular endothelial cells (HPMVECs). Cultured HPMVECs were exposed to 0.3 % CSE for 24 h, and Sirt1, CD31 mRNA, miR-34a and miR-126-3p expressions were measured by RT-PCR analysis (**a**). Sirt1 and p53 protein expression was measured by western blot analysis (**b**). CSE treatment significantly suppressed Sirt1 mRNA and protein expression and increased miR-34a and p53 protein expression. Data are expressed as mean ± SD (*n* = 6 for RT-PCR analysis, *n* = 4 for western blot analysis). *P < 0.05 versus control

We next investigated whether CSE mediates decreased Sirt1 expression and increased miR-34a expression in cultured CD34^+^ progenitor cells and HPMVECs. We found that CSE treatment significantly suppressed CD34^+^ progenitor cell proliferation at a concentration of 0.3 % (Fig. 6). Therefore, we used CSE at a concentration of 0.3 % for RT-PCR analysis of Sirt1, CD31, miR-34a, and miR-126-3p in CD34^+^ progenitor cells. CSE treatment significantly suppressed Sirt1 expression and increased the expression of p21 and miR-34a. However, no significant changes were observed in CD34^+^ progenitor cells upon exposure to 0.3 % CSE (Fig. 7). We also measured the expression of CD31 mRNA, Sirt1 mRNA, miR-34a, and miR126-3p in cultured HPMVECs exposed to 0.3 % CSE, and found almost the same gene expression patterns that in the CD34^+^ progenitor cells (Fig. 8a). We also confirmed the decreased expression of Sirt1 and increased expression of p53 protein in cultured HPMVECs exposed to CSE (Fig. 8b).

## Discussion

In the present study, we hypothesized that circulating EPC-related genes and miRNA expression could be biomarkers of the disease state in patients with COPD. We examined EPC-related gene expression in blood cells from smokers, and found that the expression of circulating CD31, CD34, Sirt1 genes, and miR-126-3p was decreased and that of miR-34a was increased in patients with moderate COPD when compared with non-COPD subjects. We also found a positive correlation between CD31 and Sirt1 mRNA expression, and confirmed the upregulation of p21 mRNA and miR-34a, and the downregulation of Sirt1 expression by exposure to CSE in cultured CD34^+^ progenitor cells. Exposure to CSE downregulated Sirt1 expression and upregulated miR-34a and p53 expression in cultured HPMVECs.

Oxidative stress can activate numerous signaling cascades that lead to lung cell apoptosis. In patients with COPD, cigarette smoke generates large amounts of free radicals, including superoxide (O^2−^), hydroxyl radical, and hydrogen peroxide (H_2_O_2_) [30]. Exposure to cigarette smoke also causes reduce of Sirt1 expression in lung tissue with concomitant elevation of matrix metalloproteinase-9 [31]. Oxidative stress is known to activate the tumor suppressor, p53, which inhibits cell cycle progression and induces apoptosis in cells with irreparable genetic damage [32]. Previous reports have shown that Sirt1 prevents cellular senescence by deacetylation and suppression of p53 [33]. In contrast, Sirt1 deficiency was reported to enhance oxidative stress-induced cellular damage and p53 acetylation [34]. It is possible that reduced Sirt1 expression under oxidative stress conditions can promote the acetylation of p53 and enhance miR-34a expression, thereby resulting in impaired angiogenesis and maintenance of lung structure with circulating EPC and COPD/emphysema. Our study demonstrates decreased expression of Sirt1 and increased expression of miR-34a, which is regulated by p53, in blood from patients with moderate COPD and in cultured CD34^+^ progenitor cells, in line with previous findings showing that oxidative stress-induced cellular damage is associated with p53 activity and Sirt1 expression [16]. Furthermore, we confirmed decreased protein expression of Sirt1 in blood cells from patients with moderate COPD (Fig. 2d). Previous studies showed that Sirt1 expression in blood cells is involved in diseases associated with systemic chronic inflammatory processes such as coronary artery disease, type 2 diabetes [35, 36]. Moreover, Mody et al., reported that decreased Sirt1 expression in pulmonary leukocyte was associated with the development of bronchopulmonary dysplasia [37]. Thus, the decrease of Sirt1 expression in blood cells from COPD patients has the potential to exaggerate inflammatory response. However, whether the decreased Sirt1 expression in blood cells from COPD patients is a result or cause of disease progression, cannot be answered by this study.

In the present study, we demonstrated increased expression of p21 mRNA and miR-34a in cultured CD34^+^ progenitor cells exposed to CSE. Both p21 and miR-34a are regulated by p53 protein, and are known to induce cellular senescence and apoptosis [38, 39]. No significant differences were observed in CD31 mRNA, CD34 mRNA, and miR-126-3p expression after CSE exposure in cultured CD34^+^ progenitor cells (Fig. 7). This suggests that decreased expression of EPC marker in patients with moderate COPD was due to the decreased number of blood cells expressing these EPC related genes, resulting from senescence and apoptosis of EPCs induced by oxidative stress from cigarette smoking. We also measured the expression of these genes in cultured HPMVECs to determine whether CSE affects lung endothelial cells, which are important in the maintenance of lung structure and participate in the pathogenesis of pulmonary emphysema [40]. We observed the same pattern of gene expression in the presence of CSE in these cells as well (Fig. 8a). Additionally, we confirmed that Sirt1 protein expression was suppressed and p53 protein expression was augmented by CSE exposure in cultured HPMVECs (Fig. 8b). Decreased expression of Sirt1 and increased expression of p53 in pulmonary endothelial cells could be also associated with the pathogenesis of COPD and emphysema, in addition to Sirt1 and its related genes in blood cells from patients with COPD.

Although the finding of a decreased numbers of circulating EPCs in patients with COPD remains controversial [4, 6, 10, 41, 42], a decrease in precursor cells might be responsible for the clinical state of patients, including disease severity, cardiovascular complications, and COPD exacerbations. Previous studies that show reduced EPCs in COPD, are related to stable patients [4, 10], and in the study by Palange, et al., current smokers, and patients with cardiovascular complications were excluded from the analysis [4]. Conversely, studies regarding circulating EPCs in COPD during exacerbations showed a significant increase in the numbers of EPCs compared to those in patients with stable COPD [42]. Our results show a decrease in the circulating cell CD31 mRNA, CD34 mRNA and miR-126-3p expression in patients with moderate COPD, and might reflect a state of relative disease stability whereas higher numbers in patients with severe COPD may be attributable to more severe systemic inflammation and the frequency of exacerbations and complications such as cardiovascular diseases. In keeping with this, we observed slight elevation in the average concentrations of CRP in patients with severe COPD. Persistent low-grade inflammation is associated with comorbidities of COPD [43], and lung inflammation may cause an increase in inflammatory cytokines in the blood, which could stimulate the circulating blood-derived endothelial progenitor cells and angiogenesis [44].

Since our study cohort had fewer patients with severe COPD that were current smokers, and a relatively large number of patients receiving ICS, we only analyzed circulating blood cell gene expression in patients with severe COPD, comparing between current smokers and ex-smokers, and between patients treated with or without ICS. However, we did not find any significant differences in gene expression between current smokers and ex-smokers among patients with severe COPD (Fig. 4a). We thus postulate that even after cessation of smoking, oxidative stress persists in activated macrophages and neutrophils, which are abundant in emphysematous lungs [45, 46]. Regarding ICS treatment, even though suppression of lung inflammation is the goal of the treatment, we observed relatively decreased Sirt1 and CD31 expression in blood cells from patients with severe COPD (Fig. 5a). This phenomenon is possibly explained by a decreased level of Sirt1 expression in lung tissue as a consequence of glucocorticoid treatment. Poulsen, et al., have previously shown that activation of the p53 pathway by glucocorticoids was associated with decreased levels of Sirt1 mRNA and protein expression in primary human tenocytes [47]. Reduced Sirt1 activity induced by ICS in COPD lungs could be related to increased p53 acetylation, and could possibly enhance p53-associated COPD/emphysema [23]. However, we cannot exclude the possibility that patients in need of ICS treatment have different EPC characteristics and reactions to oxidative stress, which affect Sirt1 and CD31expression.

Likewise, the upward shift of Sirt1 expression in patients with severe COPD could be explained by an increase in inflammatory cytokines, different smoking status or differences in treatment compared to other groups of COPD patients. A number of previous studies have demonstrated that Sirt1 is closely associated with inflammation, as Sirt1 attenuated transcriptional activation of NF-κB and decreased the production of inflammatory cytokines [48–51]. However, several previous studies have also indicated that Sirt1 could stimulate inflammatory cytokines and be associated with apoptosis resistance [52–54]. In the present study, we found a tendency for increased IL-6 expression among former smokers in severe COPD patients compared to those with moderate COPD, and a significant increase in IL-6 expression in patients with severe COPD compared to those with moderate COPD without ICS treatment (Figs. 4b and 5b). We also found a strong positive correlation among patients with severe COPD between IL-6 and Sirt1 mRNA expression in current smokers and in patients without ICS treatment (Figs. 4c and 5c). It is possible that the differences in smoking status and inhaled steroid therapy affect the upward shift of Sirt1 expression through IL-6 expression in patients with severe COPD, because of the relatively lower expression of IL-6 mRNA in ex-smokers and ICS treated patients with severe COPD (Figs. 4a and 5a). However, there is the possibility that the systemic inflammation and serum inflammatory cytokine levels due to the disease severity of COPD affected the expression of Sirt1 and its related genes in blood cells. Further studies are needed to characterize the effects of disease severity and inflammatory cytokines on Sirt1 expression in blood cells from patients with COPD.

Although there was a significant correlation between CD31 and Sirt1 mRNA expression, no significant correlation was observed between the expression of CD34 and Sirt1 mRNA. It is not surprising that the expression of CD34 and CD31 in blood cells vary from patient to patient, as CD31 is expressed in EPCs as well as platelets, and macrophages from blood cells [8]. The weak correlation between CD31 and CD34 indicates that it is difficult to evaluate EPC expression in blood cells from COPD patients by measuring the expression of a single EPC related gene in whole blood cells.

## Conclusion

In summary, our data demonstrated that EPC-related gene expression in blood cells from smokers was not associated with the severity of COPD, despite the fact that the expression of these genes was significantly decreased in patients with moderate COPD compared to that in smoking controls. The correlation between CD31 and Sirt1 mRNA expression in blood cells from smokers and the results obtained with cultured CD34^+^ progenitor cells and HPMVECs in the presence of CSE suggest the possibility that the Sirt1-p53 pathway is involved in CD31 expression of blood cells in smokers. However, further studies are necessary to clarify the pathobiological role of Sirt1-related genes in patients with COPD.

## Abbreviations

%LAA: % of low attenuation area
CD: Cluster of differentiation
COPD: Chronic obstructive pulmonary disease
CSE: Cigarette smoke extract
EPCs: Endothelial progenitor cells
ex-smokers: Former smokers
GOLD: Global Initiative for Obstructive Lung Disease
HPMVECs: Human pulmonary microvascular endothelial cells
ICS: Inhaled corticosteroids
miRNAs: Micro RNAs
PBS: Phosphate-buffered saline
Sirt1: Silent information regulator 1

## References

[1] McAllister DA, Maclay JD, Mills NL, Mair G, Miller J, Anderson D (2007). Arterial stiffness is independently associated with emphysema severity in patients with chronic obstructive pulmonary disease. Am J Respir Crit Care Med.

[2] Sabit R, Bolton CE, Edwards PH, Pettit RJ, Evans WD, McEniery CM (2007). Arterial stiffness and osteoporosis in chronic obstructive pulmonary disease. Am J Respir Crit Care Med.

[3] Kondo T, Hayashi M, Takeshita K, Numaguchi Y, Kobayashi K, Iino S (2004). Smoking cessation rapidly increases circulating progenitor cells in peripheral blood in chronic smokers. Arterioscler Thromb Vasc Biol Lippincott Williams & Wilkins.

[4] Palange P, Testa U, Huertas A, Calabrò L, Antonucci R, Petrucci E (2006). Circulating haemopoietic and endothelial progenitor cells are decreased in COPD. Eur Respir J European Respiratory Society.

[5] Michaud SE, Dussault S, Haddad P, Groleau J, Rivard A (2006). Circulating endothelial progenitor cells from healthy smokers exhibit impaired functional activities. Atherosclerosis.

[6] Takahashi T, Suzuki S, Kubo H, Yamaya M, Kurosawa S, Kato M (2011). Impaired endothelial progenitor cell mobilization and colony-forming capacity in chronic obstructive pulmonary disease. Respirology Blackwell Publishing Asia.

[7] Asahara T, Murohara T, Sullivan A, Silver M, van der Zee R, Li T (1997). Isolation of putative progenitor endothelial cells for angiogenesis. Science.

[8] Krenning G, van Luyn MJA, Harmsen MC (2009). Endothelial progenitor cell-based neovascularization: implications for therapy. Trends Mol Med.

[9] Urbich C, Dimmeler S (2004). Endothelial progenitor cells: characterization and role in vascular biology. Circ Res Lippincott Williams & Wilkins.

[10] Fadini GP, Schiavon M, Cantini M, Baesso I, Facco M, Miorin M (2006). Circulating progenitor cells are reduced in patients with severe lung disease. Stem Cells John Wiley & Sons, Ltd.

[11] Timmermans F, Plum J, Yöder MC, Ingram DA, Vandekerckhove B, Case J (2009). Endothelial progenitor cells: identity defined?. J Cell Mol Med Blackwell Publishing Ltd.

[12] Nielsen JS, McNagny KM (2008). Novel functions of the CD34 family. J Cell Sci.

[13] Kloosterman WP, Plasterk RH. The diverse functions of microRNAs in animal development and disease. Dev Cell. 2006;11:441-50. 10.1016/j.devcel.2006.09.009 17011485

[14] Gangaraju VK, Lin H (2009). MicroRNAs: key regulators of stem cells. Nat Rev Mol Cell Biol Nature Publishing Group.

[15] Bushati N, Cohen SM. microRNA functions. Annu Rev Cell Dev Biol. 2007;23:175-205. 10.1146/annurev.cellbio.23.090506.123406 17506695

[16] Zhao T, Li J, Chen AF (2010). MicroRNA-34a induces endothelial progenitor cell senescence and impedes its angiogenesis via suppressing silent information regulator 1. Am J Physiol Endocrinol Metab.

[17] Lechman ER, Gentner B, van Galen P, Giustacchini A, Saini M, Boccalatte FE (2012). Attenuation of miR-126 activity expands HSC in vivo without exhaustion. Cell Stem Cell.

[18] Tarasov V, Jung P, Verdoodt B, Lodygin D, Epanchintsev A, Menssen A (2007). Differential regulation of microRNAs by p53 revealed by massively parallel sequencing: miR-34a is a p53 target that induces apoptosis and G1-arrest. Cell Cycle.

[19] Matsushita N, Takami Y, Kimura M, Tachiiri S, Ishiai M, Nakayama T (2005). Role of NAD-dependent deacetylases SIRT1 and SIRT2 in radiation and cisplatin-induced cell death in vertebrate cells. Genes Cells Blackwell Science Ltd.

[20] Castro RE, Ferreira DMS, Afonso MB, Borralho PM, Machado MV, Cortez-Pinto H (2013). miR-34a/SIRT1/p53 is suppressed by ursodeoxycholic acid in the rat liver and activated by disease severity in human non-alcoholic fatty liver disease. J Hepatol.

[21] Mizuno S, Bogaard HJ, Kraskauskas D, Alhussaini A, Gomez-Arroyo J, Voelkel NF (2011). p53 Gene deficiency promotes hypoxia-induced pulmonary hypertension and vascular remodeling in mice. Am J Physiol Lung Cell Mol Physiol.

[22] Mizuno S, Yasuo M, Bogaard HJ, Kraskauskas D, Natarajan R, Voelkel NF (2011). Inhibition of histone deacetylase causes emphysema. Am J Physiol Lung Cell Mol Physiol.

[23] Mizuno S, Bogaard HJ, Gomez-Arroyo J, Alhussaini A, Kraskauskas D, Cool CD (2012). MicroRNA-199a-5p is associated with hypoxia-inducible factor-1α expression in lungs from patients with COPD. Chest American College of Chest Physicians.

[24] Fish JE, Santoro MM, Morton SU, Yu S, Yeh R-F, Wythe JD (2008). miR-126 regulates angiogenic signaling and vascular integrity. Dev Cell.

[25] Wang S, Aurora AB, Johnson BA, Qi X, McAnally J, Hill JA (2008). The endothelial-specific microRNA miR-126 governs vascular integrity and angiogenesis. Dev Cell.

[26] Sessa R, Seano G, di Blasio L, Gagliardi PA, Isella C, Medico E (1823). The miR-126 regulates angiopoietin-1 signaling and vessel maturation by targeting p85β. Biochim Biophys Acta.

[27] Zernecke A, Bidzhekov K, Noels H, Shagdarsuren E, Gan L, Denecke B, et al. Delivery of microRNA-126 by apoptotic bodies induces CXCL12-dependent vascular protection. Sci Signal. 2009;2:ra81–1. 10.1126/scisignal.2000610 19996457

[28] Rabe KF, Hurd S, Anzueto A, Barnes PJ, Buist SA, Calverley P (2007). Global strategy for the diagnosis, management, and prevention of chronic obstructive pulmonary disease: GOLD executive summary. Am J Respir Crit Care Med.

[29] Hanaoka M, Droma Y, Chen Y, Agatsuma T, Kitaguchi Y, Voelkel NF (2011). Carbocisteine protects against emphysema induced by cigarette smoke extract in rats. Chest American College of Chest Physicians.

[30] Pryor WA (1997). Cigarette smoke radicals and the role of free radicals in chemical carcinogenicity. Environ Health Perspect.

[31] Nakamaru Y, Vuppusetty C, Wada H, Milne JC, Ito M, Rossios C (2009). A protein deacetylase SIRT1 is a negative regulator of metalloproteinase-9. FASEB J Federation of American Societies for Experimental Biology.

[32] Roos WP, Kaina B (2006). DNA damage-induced cell death by apoptosis. Trends Mol Med.

[33] Langley E, Pearson M, Faretta M, Bauer U-M, Frye RA, Minucci S (2002). Human SIR2 deacetylates p53 and antagonizes PML/p53-induced cellular senescence. EMBO J.

[34] Furukawa A, Tada-Oikawa S, Kawanishi S, Oikawa S (2007). H2O2 accelerates cellular senescence by accumulation of acetylated p53 via decrease in the function of SIRT1 by NAD+ depletion. Cell Physiol Biochem.

[35] Breitenstein A, Wyss CA, Spescha RD, Franzeck FC, Hof D, Riwanto M (2013). Peripheral blood monocyte Sirt1 expression is reduced in patients with coronary artery disease. Stover CM, editor. PLoS ONE Public Library of Science.

[36] Song R, Xu W, Chen Y, Li Z, Zeng Y, Fu Y (2011). The expression of Sirtuins 1 and 4 in peripheral blood leukocytes from patients with type 2 diabetes. Eur J Histochem PAGEPress.

[37] Mody K, Saslow JG, Kathiravan S, Eydelman R, Bhat V, Stahl GE (2012). Sirtuin1 in tracheal aspirate leukocytes: possible role in the development of bronchopulmonary dysplasia in premature infants. J Matern Fetal Neonatal Med.

[38] Chen F, Hu S-J (2012). Effect of microRNA-34a in cell cycle, differentiation, and apoptosis: a review. J Biochem Mol Toxicol Wiley Subscription Services, Inc, A Wiley Company.

[39] Donato AJ, Morgan RG, Walker AE, Lesniewski LA (2015). Cellular and molecular biology of aging endothelial cells. J Mol Cell Cardiol.

[40] Kasahara Y, Tuder RM, Taraseviciene-Stewart L, Le Cras TD, Abman S, Hirth PK (2000). Inhibition of VEGF receptors causes lung cell apoptosis and emphysema. J Clin Invest American Society for Clinical Investigation.

[41] Takahashi T, Kobayashi S, Fujino N, Suzuki T, Ota C, He M (2012). Increased circulating endothelial microparticles in COPD patients: a potential biomarker for COPD exacerbation susceptibility. Thorax BMJ Publishing Group Ltd and British Thoracic Society.

[42] Sala E, Villena C, Balaguer C, Ríos A, Fernández-Palomeque C, Cosío BG (2010). Abnormal levels of circulating endothelial progenitor cells during exacerbations of COPD. Lung Springer-Verlag.

[43] Vanfleteren LEGW, Spruit MA, Groenen M, Gaffron S, van Empel VPM, Bruijnzeel PLB (2013). Clusters of comorbidities based on validated objective measurements and systemic inflammation in patients with chronic obstructive pulmonary disease. Am J Respir Crit Care Med American Thoracic Society.

[44] Fan Y, Ye J, Shen F, Zhu Y, Yeghiazarians Y, Zhu W (2008). Interleukin-6 stimulates circulating blood-derived endothelial progenitor cell angiogenesis in vitro. J Cereb Blood Flow Metab Nature Publishing Group.

[45] Gwinn MR, Vallyathan V (2006). Respiratory burst: role in signal transduction in alveolar macrophages. J Toxicol Environ Health B Crit Rev.

[46] Dahlgren C, Karlsson A (1999). Respiratory burst in human neutrophils. J Immunol Methods.

[47] Poulsen RC, Watts AC, Murphy RJ, Snelling SJ, Carr AJ, Hulley PA (2014). Glucocorticoids induce senescence in primary human tenocytes by inhibition of sirtuin 1 and activation of the p53/p21 pathway: in vivo and in vitro evidence. Ann Rheum Dis BMJ Publishing Group Ltd and European League Against Rheumatism.

[48] Rajendrasozhan S, Yang S-R, Kinnula VL, Rahman I (2008). SIRT1, an antiinflammatory and antiaging protein, is decreased in lungs of patients with chronic obstructive pulmonary disease. Am J Respir Crit Care Med American Thoracic Society.

[49] Caruso R, Marafini I, Franzè E, Stolfi C, Zorzi F, Monteleone I (2014). Defective expression of SIRT1 contributes to sustain inflammatory pathways in the gut. Mucosal Immunol Nature Publishing Group.

[50] Ceolotto G, de Kreutzenberg SV, Cattelan A, Fabricio ASC, Squarcina E, Gion M (2014). Sirtuin 1 stabilization by HuR represses TNF-α- and glucose-induced E-selectin release and endothelial cell adhesiveness in vitro: relevance to human metabolic syndrome. Clin Sci Portland Press Limited.

[51] Storka A, Führlinger G, Seper M, Wang L, Jew M, Leisser A (2013). E. coli endotoxin modulates the expression of Sirtuin proteins in PBMC in humans. Mediators Inflamm Hindawi Publishing Corporation.

[52] Fernandes CA, Fievez L, Neyrinck AM, Delzenne NM, Bureau F, Vanbever R (2012). Sirtuin inhibition attenuates the production of inflammatory cytokines in lipopolysaccharide-stimulated macrophages. Biochem Biophys Res Commun.

[53] Wendling D, Abbas W, Godfrin-Valnet M, Guillot X, Khan KA, Cedoz J-P (2013). Resveratrol, a sirtuin 1 activator, increases IL-6 production by peripheral blood mononuclear cells of patients with knee osteoarthritis. Clin Epigenetics BioMed Central.

[54] Niederer F, Ospelt C, Brentano F, Hottiger MO, Gay RE, Gay S (2011). SIRT1 overexpression in the rheumatoid arthritis synovium contributes to proinflammatory cytokine production and apoptosis resistance. Ann Rheum Dis BMJ Publishing Group Ltd and European League Against Rheumatism.

